# Cell Guidance by 3D-Gradients in Hydrogel Matrices: Importance for Biomedical Applications

**DOI:** 10.3390/ma2031058

**Published:** 2009-08-25

**Authors:** Tessa Lühmann, Heike Hall

**Affiliations:** Cells and BioMaterials, Department of Materials, HCI E415, ETHZ, Wolfgang-Pauli-Strasse 10, CH-8093 Zürich, Switzerland; E-Mail: tessa.luehmann@mat.ethz.ch (T.L.)

**Keywords:** extracellular matrix, integrins, gradients, hydrogels, cell guidance, haptotaxis

## Abstract

Concentration gradients of soluble and matrix-bound guidance cues in the extracellular matrix direct cell growth in native tissues and are of great interest for design of biomedical scaffolds and on implant surfaces. The focus of this review is to demonstrate the importance of gradient guidance for cells as it would be desirable to direct cell growth onto/into biomedical devices. Many studies have been described that illustrate the production and characterization of surface gradients, but three dimensional (3D)-gradients that direct cellular behavior are not well investigated. Hydrogels are considered as synthetic replacements for native extracellular matrices as they share key functions such as 2D- or 3D-solid support, fibrous structure, gas- and nutrition permeability and allow storage and release of biologically active molecules. Therefore this review focuses on current studies that try to implement soluble or covalently-attached gradients of growth factors, cytokines or adhesion sequences into 3D-hydrogel matrices in order to control cell growth, orientation and migration towards a target. Such gradient architectures are especially desirable for wound healing purposes, where defined cell populations need to be recruited from the blood stream and out of the adjacent tissue, in critical bone defects, for vascular implants or neuronal guidance structures where defined cell populations should be guided by appropriate signals to reach their proper positions or target tissues in order to accomplish functional repair.

## 1. Introduction 

Concentration gradients of soluble growth factors or matrix-immobilized guidance cues play fundamental roles during embryogenesis [1] and in a number of physiological processes in the adult; including recruitment of different cell types in wound healing [2,3,4,5], homing of immune cells [3,6] and nerve regeneration [7,8,9]. Both, chemical and physical factors can influence the orchestrated orientation and migration of cells towards a specific target location [10,11,12,13,14,15,16]. *In vivo*, the developing embryo or a growing child displays defined polarity; namely: head and foot, dorsal and ventral axis that, in combination with gravitational forces lead to directed cell orientation and cell growth [5,6,17,18,19]. For synthetic tissue substitutes the difficulty lays in producing molecular gradients of chemical or physical guidance cues on top or within a homogeneous scaffold material. Such a scaffold material is produced and analyzed on the macromolecular scale, whereas it needs to modulate cellular responses on the molecular scale, since cells sense their 2D- and 3D-environment by forming transient contacts to their surrounding extracellular matrix. Therefore implementation of soluble or covalently attached cell guidance cues within a biomaterial imposes a large challenge to biomaterials research and production. This current review will first illustrate the importance of the native extracellular matrix for cellular functions, their potential replacement by 3D-hydrogel matrices and then give several examples on how cell guidance cues can be implemented onto and into 3D-hydrogel matrices in order to improve biomaterial functions by inducing directional cell growth.

### 1.1. Native Extracellular Matrix

The native extracellular matrix (ECM) refers to a complex network of molecules that provide 2D- or 3D-mechanical support for cells, serves as a barrier between different compartments or cell types and provides guidance cues during development and wound healing or tissue repair. On the individual cell basis ECM induces cell polarity, allows or inhibits cell adhesion, promotes or slows down migration and induces cell and tissue differentiation and might also induce receptor mediated apoptosis [20,21,22]. The ECM is composed of chemically very different macromolecules that are assembled into organized structures remaining in close association with the surface of the cells that secreted them; being predominantly fibroblasts, osteoblasts and chondrocytes. The main components are space filling proteoglycans, containing collagen fibers and non-collagenous glycoproteins such as elastin. Integrated into this hydrogel-like matrix are signalling molecules being growth factors, cytokines and hormones [23,24]. The hydrogel-like ECM resists compressive and shear forces exerted on the matrix but permits rapid diffusion of nutrients, metabolites and hormones between the blood stream and tissue cells. The ECM occurs in many different forms depending on the requirements of the host tissue. In many cases it is a 3D-strucuture that surrounds tissue cells maintaining the tissue specific 3D-architecture. In other cases the ECM forms flexible sheet-like structures between 40-120 nm thickness that serve as solid support layers composed of network forming laminin/ entactin complexes, type IV collagen and heparan sulfate proteoglycans. The sheet-like ECM is called basal lamina and is especially found around blood vessels, muscle and nerve fibers or to separate different compartments within one organ e.g. between α and β cells in the pancreas [25]. The ECM is tissue specific and the components self assemble to form spontaneous 2D- or 3D-structures under physiological conditions. Moreover, the ECM is not a static structure but is constantly remodelled by cellular activity. Many cell types including fibroblasts, endothelial cells, osteoclasts, macrophages and others secrete proteolytic enzymes such as matrix metalloproteinases and/or serine proteases that degrade ECM components at very specific locations thus allowing cell migration through the ECM and exposing buried cell binding sites [26]. This feature is especially important in tissue growth and wound healing/regeneration processes or after insertion of an implant; as it indicates that the tissue cells themselves are able to design and modify already existing, initially preliminary or transient matrices in order to synthesize their final and highly specialized tissue-specific ECM.

#### 1.1.1. Cells interacting with the ECM

All body cells, except the blood cells, interact directly and in a very specific manner with their surrounding ECM. Specific receptor-ligand contacts are established that enable mutual communication between the ECM and the interior of the cell thus regulating matrix assembly, specific remodeling and local removal or disassembly of the matrix [26]. Cells react to distinct physical and biochemical characteristics of the ECM. Although it is well known that cells respond in different fashion to distinct matrix molecules, (i.e. composition of the matrix), they also recognize differences in pliability of the matrix, tension forces, and the spatial nature of the matrix (2D versus 3D) [26,27,28,29,30,31]. These responses result in a variety of adhesion structures formed. When fibroblasts are cultivated on 2D-surfaces, they tend to spread in a flattened morphology whereas fibroblasts surrounded by a 3D-fibrous matrix assume an elongated cell shape that seems to mimic fibroblastic cells *in vitro* [30]. Apparent cell morphology is a direct consequence of the ability to form specific cell-to-matrix adhesion complexes. On 2D-surfaces focal complexes are formed that require binding to only one ECM ligand. Focal contacts first mature to focal adhesions allowing cell contractility and then further to fibrillar adhesions that require fibronectin in addition to the initial ECM ligand [30,31]. Fibrillar adhesions are elongated structures that allow cell alignment with fibers of the extracellular matrix, cell contractility and occur only on pliable 2D-substrates or in 3D-matrices. Most mature matrix contacts are formed only in 3D-matrices and are therefore collectively called 3D-adhesions [30]. Cell-to-matrix contacts are mainly formed between different integrins assembled into giant transmembrane protein complexes that regulate and specify their ligand binding affinity as well as matrix assembly. In addition integrins connect the ECM intimately to the actin cytoskeleton e.g via talin or kindlin or to intermediate filaments leading finally to information transmission to the nucleus where new proteins can be synthesized [32,33,34,35,36]. Integrins are transmembrane heterodimeric glycoproteins consisting of one α (~ 130 to 160 kDa) and one β subunit of ~ 110 kDa forming at least 24 integrin-heterodimers known in humans. Many integrins require divalent cations (Ca^2+^, Mg^2+^ or Mn^2+^) for structural integrity and ligand binding as well as activation through cluster formation in order to be fully functional [33,34,37,38,39,40]. Although individual integrin-ECM-ligand interactions are of low affinity (between 10^6^ to 10^9^ M), the high integrin abundance on the cell surface and their clustering potential produces very strong cell-to-matrix interactions allowing mechanical force transmission and cell migration [26,28,29]. Many cell-to-matrix contacts are formed and enable the cell to respond to their immediate 2D- or 3D-environment. These contacts are transient and strongly regulated such that a migrating cell is able to form new contacts at the leading edge whereas at the back end cell-to-matrix contacts are released. Often formation of cell-to-matrix contacts can be correlated with directed cell migration towards a specific target organ. Especially neutrophiles, endothelial cells or nerve growth cones orient along molecular guidance cues implemented within the native extracellular matrix [4,5,6,41,42,43].

#### 1.1.2. Gradients for cell guidance in the native ECM

Three major mechanisms are described that direct cells along a gradient of attractive guidance cues: haptotaxis is defined as directed movement of cells along the direction of a gradient of matrix-immobilized ligands [44,45]. Cells preferentially move towards the direction of higher adhesiveness on substrates with increasing density of ligands, with a velocity which is tunable by the slope of the ligand gradient [46,47,48,49]. In contrast directional cell migration in response to a concentration gradient of soluble chemoattractants is defined as chemotaxis [4,6,26,43,50]. Directed cell orientation induced by mechanical forces exerted through the matrix or by shear stress in blood vessels is referred to as mechanotaxis [27,30,51]. Here cellular responses are direct consequences of differential mechanical properties of the surrounding matrix suggesting that cells sense the stiffness of their 2D-substrate or surrounding 3D-matrix and respond accordingly [27,28,29,52]. From now on this review will further concentrate on chemotactic and haptotactic cell guidance as it might be relevant for cellular guidance on/into biomedical implant materials.

The native ECM contains soluble and matrix-bound biological signals that guide cells towards their natural targets. Often the native ECM is filled with interstitial fluid that allows gradient-type distribution of soluble signaling molecules such as growth factors, chemokines, hormones, gases and nutrition. Diffusion speed and distance is limited by the molecular dimensions of the ECM (pore size, interconnectivity, fiber diameter and interactions with the fibers), the diffusion properties of the diffusing molecules and the concentration gradient(s) in the tissue [53,54]. The larger the molecule, the more important fluid convection becomes (relative to diffusion). In the physiological range of convective interstitial flows (~0.1–1.0 μm/s) [55,56] large molecules such as antibodies or growth factors (Mw: above 35 kDa) are influenced strongly by convection, whereas small solutes (oxygen, glucose, Mw below 1,000 Da) rely mainly on diffusion [53,57,58]. Because the actions of chemokines and growth factors depend largely on their local gradients relative to the cell in addition to the absolute amounts, interstitial flows are important regulators of tissue behaviour [5,41,42,56]. The effects of interstitial flow can be considerable when numerous molecules are involved, as shown recently when endothelial cells were embedded into fibrin matrices. Slow interstitial flow of 2–10 μm/s was observed to act in synergy with matrix-bound vascular endothelial growth factor (VEGF) to enhance capillary morphogenesis [59]. Moreover, gradients of growth factors along the length of a cell can be as small as <1% but are still able to trigger cell responses [60,61,62,63]. Natural ECM does not only contain soluble but also matrix-bound cellular guidance cues. Especially heparin-binding proteoglycans present in large amounts within the ECM serve as storage and release system for many growth factors e.g. for platelet derived growth factor (PDGF), fibroblast growth factor (FGF)-2, VEGFs, nerve growth factor (NGF) and glial derived growth factor (GDNF) all containing heparin-binding sequences thus allowing direct binding to the sulphated glycosaminoglycan chains of proteoglycans. Through local changes in pH or salt growth factors can be either stored or released from the matrix and therefore become available for cells [23,24,64,65,66,67,68]. Another mechanism to enable activation of ECM-bound growth factors was shown for FGF-2 that forms a ternary complex between FGF-2, a sequence of at least 12 saccharides containing sulfate groups at C2 of iduronic acid as well as at C6 of glucosamine [69] and one of the four structurally related high-affinity FGF receptors [70,71]. Through cell migration, accompanied with local matrix degradation cells come in close proximity to matrix stored FGF-2, form active ternary complexes and FGF-2 can induce its mitogenic and anti-apoptotic functions [23]. In addition, it was shown that individual growth factors occur in differently spliced isoforms containing heparin-binding sites or not. This is the case for large VEGF-isoforms and VEGF _121_, respectively [72]. When the site of VEGF expression and the localization of the VEGF proteins were compared, it was demonstrated that VEGF _121_ diffused over considerable distance within tissues, whereas the large heparin-binding VEGF isoforms formed a steep extracellular VEGF gradient. Ruhrberg and colleagues concluded that diffusible VEGF _121_ reached the endothelium over large distances and stimulated continued proliferation of endothelial cells, which in turn increased vessel diameter, whereas matrix-bound VEGF gradients were so steep that vessels branched excessively [64,73]. Therefore the equilibrium between matrix-bound and soluble growth factor(isoforms) seem to regulate the normal endogenous distribution, where stable growth factor gradients can be maintained by retention of this growth factor close to the site of production to build an extracellular gradient [24,41].

#### 1.1.3. Cellular responses to gradients

Different cell types are able to follow soluble or matrix-bound gradients of guidance cues. Often neutrophils were studied that follow concentration gradients to migrate towards a site of inflammation [74]. Soluble gradients of cyclic adenosine monophosphat (cAMP), different interleukins, antibodies or cytokines were generated in Boyden chamber–type experiments to analyze neutrophil chemotaxis *in vitro* [75,76]. Many studies describe neuronal growth cones exploring their 2D-substrates following haptotactic or soluble attractive molecular guidance cues [43,77,78]. Also the development of capillary sprouts towards a gradient of matrix-bound VEGF and/or hypoxia was described in detail [5,41,42]. All cell types usually follow a three-step program: directional sensing, motility and polarization [43,74,79]. Directional sensing refers to the ability of a cell to transduce a shallow externally presented gradient of guidance cues into a significantly steeper intracellular gradient. It is suggested that signal amplification occurs through a local-excitation and global-inhibition mechanism in which receptor mediated signals generate two types of signals: a local production of the signaling molecule combined with overall degradation of this molecule. Through this process the cell is able to reflect the steepness and amplify the external gradient of guidance cues by creating a spatial separation of intracellular signals thus creating a cellular asymmetry [43,80]. Cell motility is characterized by a spatiotemporal coordination of the cytoskeleton and formation of cell-to-matrix adhesion in relation to the stiffness of the 2D-substrate and/or the pore size of 3D-matrices allowing traction forces for directed net movement [43,81,82]. Lauffenburger et al have shown that fibroblasts exert traction forces on the matrix in an epidermal growth factor-dependent manner playing important roles in force generation and transmission during cell migration [47,48,83,84]. Gradient sensing and generation of traction forces result in the polarization of the cell having a defined front and rear end.

In order to maintain forward and directed movement a cell enters the so called ‘migration cycle’ which consists of repeating intervals with a life time of <30 s where self-organizing cytoplasmic structures (lamellipodia or filopodia) are produced into the direction of the attractive guidance cue [85]. These cytoplasmic extensions display high sensitivity towards extracellular (gradient)stimuli that can often be seen as extensive membrane ruffles at the front. Such membrane ruffles contain a high concentration of receptors at the leading edge suggesting high asymmetric sensitivity to external stimuli [43,74,86]. The membrane protrusions degrade 3D-matrices when necessary and form transient integrin-mediated matrix contacts and connect to the actin cytoskeleton therefore they serve as traction sites for forward migration whereas microtubules mediate cell contraction and removal of matrix-contacts at the rear end [79,83,84]. Maintenance of polarization occurs by different feedback loops that sense the cell-to-matrix contacts leading to differential gene expression. Many genes including PI3K, Cdc-42, Rac-1 and MAPK are involved in maintenance of the polarization and have been reviewed recently [74,81,87,88,89]. Directed cell movement was modeled using different models including integrin-dependent matrix interactions and direction and speed was modelled in 3D-matrices implementing the mechanical forces and viscosity of the ECM [49,90,91]. The models predict a biphasic behaviour of cell speed in 3D matrices. The biphasic behaviour suggests that maximum speed is obtained at intermediate values of adhesivity, and that at extreme values the cells show little or no motility therefore the model predicts that cell speed is sensitive to the number of ligands in the ECM [90].

### 1.2. Gradients for Biomedical or Technical Applications

**Figure 1:**
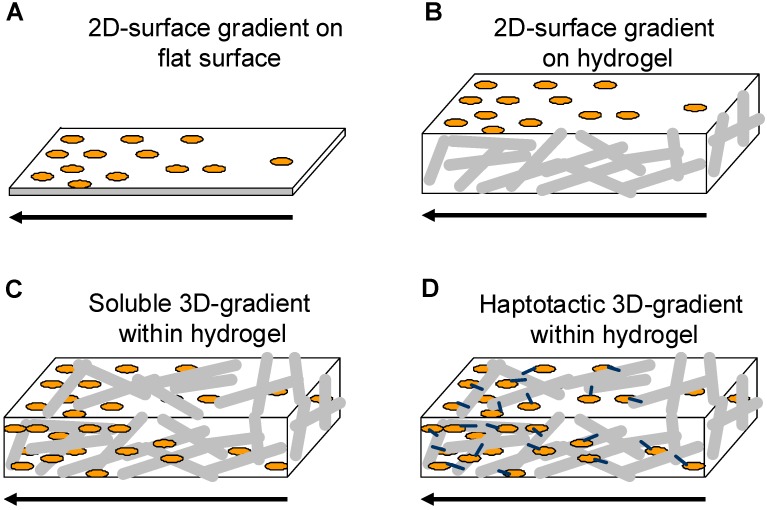
Schematic of 2D and 3D-gradient matrices.

When developing new materials with potential applications in biomedical areas, material surfaces are in direct contact with the surrounding tissue and therefore need to be biocompatible and should ideally induce specific cellular responses. In recent studies many approaches were developed that gradually varied surface properties (chemical and physical) in one dimension (Figure 1A). Such gradient surfaces expose a selected property in order to screen biological responses without any boundary conditions. Therefore chemical and biological surface gradients are interesting tools for basic and applied studies that will lead to optimal surface characteristic for a selected application. These gradient type of surfaces have been extensively studied and reviewed recently in [10,15].

Another biomedical application of gradients would be to direct cell growth on implant surfaces in order to improve regenerative or healing/integration processes or guide cells into the core of porous or fibrous implants. Such applications would require a combination of physical and biological/chemical guidance structures that simulate functions of the native ECM allowing not only implant functionality but inviting cells to differentiate and behave according to their respective functions. Materials might be modified such that they release a soluble gradient of attractive guidance cues or 2D- and 3D- materials can be chemically modified to expose a gradient of fixed guidance cues comparable to haptotactic gradients of matrix-bound growth factors being responsible for directed cell migration in native tissues.

**(**A) 2D-surface gradient: guidance cues are released in soluble form or haptotactic cues are surface attached. Cells can be placed on top of the gradient and e.g. cell alignment or migration can be studied on continuously changing conditions. (B) 2D-gradients on hydrogels: haptotactic gradients of guidance cues are surface attached on 3D-hydorel matrices. Cells are cultivated on top of the gradient and e.g. cell alignment or migration can be studied. (C) Soluble 3D-gradients within hydrogels: soluble guidance cues are provided and allow cell-cultivation on top or within the 3D-hydrogel matrix. (D) Haptotactic 3D-gradients within hydrogel matrices. Hydrogel matrices contain covalently fixed gradients of growth factors or specific adhesion sequences that guide the cells. Cell alignment and migration towards the concentration gradient in 3D can be studied.

Most studies performed to establish haptotactic gradients designed 2D-surface gradients of bioactive guidance cues that are immobilized by photoinitiated- or heterobifunctional coupling reactions [92,93,94,95,96,97] microstamping gradient pumping [98] and microfluidic techniques [99,100]. Besides chemical guidance topographical structures were shown to direct cells such as rat hippocampal neurons on silicon oxide surfaces or primary fibroblasts cultured on nanogrooved patterns [101,102,103,104]. In addition differential substrate adhesiveness was shown to attract and direct cell growth of fibroblasts, myocytes and neuronal cells [52]. All approaches enable the establishment of defined gradients that allow studying cellular responses on 2D-surfaces, but unfortunately the native three-dimensional environment cannot be addressed with those approaches although highly desirable to mimic and understand the native cellular 3D-environment.

### 1.3. Hydrogel Matrices as Replacements for Natural Extracellular Matrices

Hydrogel matrices are highly swollen three-dimensional fibrous structures consisting of linear or branched monomers that are connected by covalent or associative bonds to form a three dimensional fibrous network. Hydrogel matrices can be grouped into hydrogels formed from purely synthetic monomers (e.g. PEG or polyacrylamide-based or structures), from monomers with natural origin such as collagen, fibrin, agarose or combinations of both (for review: [105,106,107,108,109] The mechanical properties of hydrogels can be adapted by varying the cross-link density or the molecular weight and branching of the monomers such that they match the tissue they need to substitute or replace. Polyacrylamide hydrogels, for example, can be cast as thin films showing mechanical strength between 100 to 10,000 Pa [110,111,112]. Polyester urethane matrices were shown to meet mechanical properties of most tissues of the human body [113]. For soft tissue replacement such as during wound healing collagen- or fibrin based hydrogels were investigated and used [114]. Fibrin and collagen hydrogel matrices provide different mechanical properties and pore sizes when compared to synthetic polyethylene-glycol (PEG)-based hydrogels. Collagen matrices were found to have the largest pore sizes of 7.4 μm followed by fibrin with 0.6 μm, whereas the pore size in PEG-based hydrogels was very small (0.025 μm) [115]. Inversely related were mechanical properties with 290 Pa for PEG-based, 27 for fibrin and 7 Pa for collagen matrices, when comparing all at 2 mg/mL solid content [115,116]. These data show that hydrogel matrices contain a high percentage of water, permitting diffusion of gases as well as nutrients, proteins and signalling molecules [53,57]; all together mimicking some key features of the native ECM. When produced for tissue engineering purposes 3D-hydrogel matrices provide a versatile platform for molecular interactions with target cells or tissues since they can be modified with biologically active signals such as adhesion sequences or growth factors [108,117]. Moreover, hydrogel matrices are usually composed of soluble precursor solutions that can therefore be applied as stand alone matrices for complex implant geometries or as interpenetrating matrices within shape giving implants. Hydrogels often polymerize under very mild conditions. In addition to their structural similarity to the native ECM, hydrogel matrices can be used as depots for drugs that are released by hydrolytic degradation of the hydrogel or on specific cellular demand [108,118,119] reviewed in [109]. Hydrogel release systems have been explored for delivery of bFGF-2 from peptide amphiphiles to increase subcutaneous neovascularization [120]. Moreover, native hydrogel matrices such as fibrin, chitosan, hyaluronan, gelatine or collagen were used in various applications to increase wound repair and angiogenesis by releasing growth factors and other bioactive molecules [108,117,121] reviewed in [61,121,122,123].

#### 1.3.1. Gradients in 3D-hydrogel matrices

Hydrogel matrices were shown to be versatile three dimensional structures that are used for many tissue engineering and drug delivery applications. So far hydrogels were produced in a way such that the physical and chemical/biological compounds are homogeneously distributed. In order to implement directionality into hydrogel matrices three different ways have been explored: i) soluble gradients within 3D-hydrogel matrices, ii) 2D-gradients on top of 3D-hydrogels and iii) 3D-haptotactic gradients within hydrogel matrices that direct cell alignment and migration to finally reach a specific target.

##### Soluble gradients within 3D-hydrogel matrices

Several approaches have been explored to produce gradients of soluble guidance cues (Figure 1C; Table 1). Most studies use microfluidic devices to determine effects of gradients of guidance cues on different cell types such as endothelial cells, fibroblasts and neurons [51,77,78,124,125,126]. Microfluidic devices are very convenient as they save material, are very controllable and soluble gradients can easily be produced. However, they face the disadvantage that it is very difficult to produce 3D-hydrogel matrices within the small dimensions of microfluidic chambers as viscous hydrogel precursor solutions seem to be difficult to handle. Only few studies describe the formation of 3D-hydrogel matrices containing soluble gradients of growth factors on a larger scale. The study described by Knapp *et al*. uses 3D-fibrin or collagen matrices placed into a two-chamber system, which is separated by a Teflon plate [127]. One of the hydrogel matrices contains different concentrations of soluble glycine-arginine-glycine-aspartate-serine-proline-(GRGDSP)-peptides, which were induced to diffuse into the non-peptide containing hydrogel after removal of the Teflon plate. Gradients of soluble GRGDSP formed and were found to be stable for 24 h. Cell alignment and migration towards the high concentration of the soluble guidance cue was assesses using human fibroblasts that were embedded into the 3D-matrices [127]. This assay provides one possibility to formulate gradients of guidance cues under physiologically more relevant conditions as compared to simple surface gradients established in Boyden chamber-like experiments [75,76]. Another study demonstrated generation of growth factor gradients via microsphere delivery when embedded into biopolymeric scaffolds [128]. The idea is that polymeric polylactic-co-glycolic acid (PLGA) and silk fibroin microspheres were fabricated and placed in a gradient manner into a cylinder shaped-agarose or aqueous silk hydrogel. The microsphere gradients within these hydrogels were produced by a gradient maker. Microspheres containing bone morphogenetic protein 2 (rhBMP-2) and/or insulin-like growth factor I (rhIGF-I) released their content over time thus generating linear gradients of rhBMP2 and/or rhIGF-1, respectively. Human bone marrow-derived mesenchymal stem cells (hMSCs) were embedded into the scaffolds and their osteochondral differentiation was analyzed after several weeks. This microsphere/scaffold system offers new options for the delivery of single or multiple growth factors with spatial control in a 3D-culture environment.

Several groups have developed culture systems to analyze directional angiogenesis even into complex tissues or organs e.g. [125]. Here a macromolecular fluid device was developed that allowed cultivation of 3D-tissues in order to be able to monitor outgrowth of new blood vessels as a result of a gradient stimulus. The device consisted of a round culture chamber that was connected to a perfusion system that allowed production of soluble gradients of growth factors. This culture system has been used for the analysis of directed blood vessel growth in embryonic mouse kidneys or in clusters of differentiating stem cells. All approaches mentioned above use soluble gradients of guidance cues that need to be maintained in a very stable way over the entire cultivation period. Therefore approaches that generate matrix-immobilized gradients of haptotactic guidance cues were developed.

##### 2D gradients on 3D-hydrogel matrices

Several studies have explored the possibilities to generate haptotactic gradients on top of different types of hydrogels (Figure 1B; Table 1). These approaches combine tunable mechanical properties of the hydrogel as cell substrate with relative ease in generating gradient features that direct cell growth. Inkjet printing of macromolecules onto hydrogels have been used to study stem cell differentiation [129]. Poly-acrylamide-hydrogels were produced to carry printed gradients of FGF-2, ciliary nerotrophic factor (CNTF) or fetal bovine serum (FBS). Primary fetal neural stem cells were seeded on top of these gradients and increasing differentiation towards a glial fibrillary acid protein (GFAP)-positive phenotype was observed in correlation with the increase in CNTF gradients. On FBS gradients neural stem cells differentiated towards smooth muscle cells whereas on gradients of FGF-2, no differentiation was observed. These studies demonstrate that differentiation of neural stem cells along matrix-bound gradient cues can be achieved and that such techniques are relatively easy to obtain spatial control of matrix features. Campbell *et al*. used linear inkjet printed gradients of FGF-2 on fibrin hydrogel films that were stable for more than 10 days under tissue culture conditions [130]. Human MG-63 osteosarcoma cells were analyzed and showed increased proliferation rates with increasing concentrations of FGF-2 gradients.

##### Covalent 3D-haptotactic gradients within hydrogel matrices

As hydrogel matrices have some structural similarity to the native extracellular matrix they might also be used as 3D-guidance structures for directed cell growth. Very few studies demonstrated 3D-gradients of guidance cues or growth factors covalently immobilized into 3D-hydrogel matrices (Figure 1D; Table 1). Among these studies one has to differentiate between the culture conditions for cells: some studies produce 3D-gradients of guidance cues or growth factors, however culture the cells to be analyzed on top of the gradient such that the cells are only exposed to a gradient substrate. This approach often holds true for synthetic hydrogel matrices as they show extensive swelling behavior or gradient immobilization was performed by photopolymerization, both not easily compatible with cell cultivation within the hydrogel matrices. When native hydrogel matrices and mild gradient immobilization techniques are used, cells can be entirely integrated into gradient matrices thus simulating more native culture conditions.

Hydrogels of (meth)acrylated polyethylene glycol precursors were formed that contained covalently linked 2D- or 3D-gradients of RGDSC-peptides [131]. The hydrogels were preformed and later perfused with RGDSC-peptides that were then thiolene-photocoupled. The gradients were shown to be linear over a distance of 2.5 mm and within a hydrogel matrix of ~230 μm thickness. Another study produced fibrin matrices in a preformed mould, which could then be perfused with a sulfo-LC-SPDP coupling agent [93]. The perfusion was performed in the presence of sucrose that slowly replaced the buffer solution; thus the resisting time of the coupling agent within the fibrin matrix provided a gradient of the bifunctional coupling agent that was then coupled to a thiol-containing fluorophore. The gradient linearly increased over 8 mm. Unfortunately neither study provided any data on cells recognizing such gradients and align or migrate towards the higher concentration of the guidance cue.

When RGDS-peptide gradients in PEG-based hydrogels were formed by a gradient maker [132] the RGDS-peptide was immobilized within the PEG-hydrogel by photopolymerization. A linear gradient occurred over 5 cm of the hydrogel and the slope depended on the final concentration of the RGDS-peptide. Human fibroblasts were cultivated on top of the gradient and showed maximal alignment (54%) after 4 days with the RGDS-peptide immobilized between 0 and 1 μmol/mL. Moreover human fibroblasts migrated further towards the high concentration of RGDS-gradients and this migration behavior depended on the slope of the RGDS-gradient. bFGF gradients (0-50 nmol/mL) were immobilized in a similar manner after reaction with acryloyl-PEG-NHS [133]. The hydrogels were formed by combining acryloyl-PEG-bFGF, PEG diacrylate, acryloyl-PEG-RGDS in the presence of long-wavelength ultraviolet light and a photoinitiator (2,2-dimethyl-2-phenyl-acetophenone in *N*-vinyl-pyrrolidone). bFGF-modified hydrogels with RGD adhesion sites were evaluated for their effects on vascular smooth muscle cells cultivated on top of the hydrogels and showed alignment on hydrogels modified with a bFGF gradient in the direction of increasing tethered bFGF concentrations as early as 24 h after seeding. Smooth muscle cells also migrated up the concentration gradient of bFGF as compared to control hydrogels with and without a constant bFGF concentration [133].

There are only few reports that describe cell cultivation within 3D-hydrogel matrices containing haptotactic gradients within hydrogel matrices [8,62,134]. These studies use different techniques to establish their gradient hydrogel matrices. Dodla used preformed agarose hydrogels that can be perfused with laminin-1 and later photolpolymerized to form covalently-fixed gradients of laminin-1 [134]. Different gradient steepness was obtained by using increasing concentrations of laminin-1 prior to diffusion through the agarose matrix. Chicken dorsal root ganglion neurons were placed in these gradient matrices and neurite extension was determined revealing significantly higher neurite extension rates in gradient matrices as compared to homogeneous laminin-1-containing agarose hydrogels [134]. Moerover, these lmainin-1 gradient matrices were used *in vivo* to increase rat sciatic nerve regeneration [8]. Gradient laminin-1 matrices were placed within 20 mm sciatic nerve gaps and analyzed after 4 month *in vivo* for axon density, amount of myelination and re-gain of function. Interestingly the study showed clearly that laminin-1-gradient matrices only in combination with a gradient of nerve growth factor (NGF) promoted much better nerve regeneration as compared to hydrogel matrices containing homogeneous concentrations of laminin-1 or NGF, or laminin-1 or NGF-gradients alone combined with a homogeneous concentration of the respective other molecule [8].

**Figure 2:**
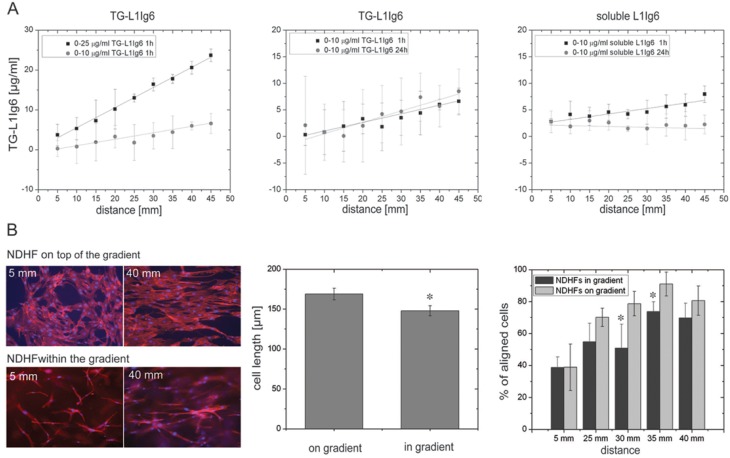
Haptotactic 3D-gradients within fibrin hydrogel matrices guide cells. Adapted from Ref. [62].

Another study established covalently immobilized gradients of the 6^th^ Ig-like domain of cell adhesion molecule L1 (L1Ig6) in a 3D-fibrin matrix. L1Ig6 was previously shown to act as a ligand for αvβ3 and α5β1 integrins found on many cell types including endothelial cells and neurons [135,136,137]. The design of a hybrid protein, consisting of L1Ig6 and a transglutamase factor XIIIa substrate sequence (NQEQVSPL; named TG-L1Ig6) at the *N*-terminus enabled covalent incorporation of TG-L1Ig6 into fibrin matrices [138,139]. Linear gradients of TG-L1Ig6 were established by a piston-driven gradient mixer and were shown to be stable for at least 24 h whereas gradients from soluble included guidance cues disappeared completely (Figure 2A). Fibroblast alignment along the gradients was observed when cultured on top and within TG-L1Ig6 gradient matrices (Figure 2B).

(A) Covalent gradients of TG-L1Ig6 were performed with different final concentrations revealing different slopes of the gradients (left). Covalent gradients of TG-L1ig6 were found to be stable after 24 h (middle) whereas gradients of soluble L1Ig6 disappeared (right). (B) Normal dermal human fibroblasts adhered on top and within 3D-fibrin matrices containing a gradient of 0-10 μg/mL TG-L1Ig6 (left). Cell length was found to be different for cells cultivated on top or within the gradient matrices and correlated nicely with cell alignment over the length of the gradient (middle). The fibroblasts aligned with the direction of the gradient (increase between 5 and 40 mm from the beginning of the 0-10 μg/mL gradient of TG-L1Ig6; right).

An increase of 0.2 µg TG-L1Ig6/mL per mm matrix was observed to be sufficient to achieve cellular response, corresponding to a concentration change of <1% per cell. When fibroblasts were cultured within the TG-L1Ig6-gradient matrices the number of aligned cells decreased by 20-30% in the middle of the gradient in comparison to cells cultivated on top of the gradient. In parallel the average cell length of fibroblasts was observed to decrease by ~ 13% within the gradient matrix compared to fibroblasts cultured on top of the gradient matrix. In contrast to fibroblasts endothelial cells did not show any alignment when cultivated on top of TG-L1Ig6 gradient matrices. The study indicates that different cell types exposed to gradients of matrix-bound TG-L1Ig6 are able to respond differentially to 2D or 3D-environments suggesting that also artificially produced 3D-hydrogel matrices contain very specific biological information that can be recognized and processed and the cells respond accordingly.

### 1.4. Summary

This review points out the importance of the 2D- and 3D-extracellular surrounding of tissue cells that is important not only for structural support but also for cell guidance during different biological processes during embryonic development, in the adult and during wound healing and tissue repair processes. The extracellular matrix consists of scaffold macromolecules embedded with soluble and matrix-bound biological active molecules (growth factors, cytokines, hormones, nutrition, gases etc.) that can be homogeneously distributed but very often these molecules form local gradients. These gradients can be formed by long-distance diffusion of soluble molecules through the interstitial fluids or by secretion of matrix-associated molecules that remain in close proximity with the cells that secreted them. Small molecular gradients (<1%) can be recognized by different cells in a differential way and are amplified within the responsive cell to induce the appropriate response such as directed cell growth, alignment or migration towards high concentrations of the guidance cue. 3D-hydrogel matrices are described as potential synthetic (transient) substitutes for the native ECM as they combine several key features: structural support, high permeation capacity for gas and biomolecules therefore suggesting the use of gradients for cell guidance within 3D-scaffolds might be an option for improvement of biomedical implants.

Three main groups of gradients of cellular guidance cues on/in 3D-hydrogel matrices produced by various techniques were discussed: gradients of soluble growth factors within 3D-hydrogel matrices, immobilized 2D-gradients on top of 3D-hydrogels and 3D-haptotactic gradients within hydrogel matrices (see also Table 1). All gradients share the common feature of directing cellular responses towards the increasing concentrations of attractive guidance cues and are therefore very interesting tools to induce directional biological responses. However different types of gradients face severe limitations for long term use. When using soluble gradients of guidance cues the gradient needs to be maintained over the entire cultivation period in order to generate stable conditions for cell guidance. For *in vitro* studies these conditions might be easily controllable but such gradients would probably disappear fast when implanted into a living organism as interstitial and blood flow would lead to fast diffusion of the guidance cue out of the 3D-hydrogel matrices. Therefore approaches that generate matrix-immobilized gradients of haptotactic guidance cues were explored. Guidance cues have been covalently-immobilized on the surface of 3D-hydrogel matrices. Such gradient matrices are more stable as compared to matrices containing gradients of soluble guidance cues however the three-dimensionality of native tissue is not maintained. Cells cultivated on top of these gradients are exposed to it only from their ventral side thus reducing the system to a 2D-situation. When cells are cultivated within hydrogel matrices containing gradients of matrix-bound guidance cues a more native culture situation is obtained as the entire cell is exposed to the gradient of guidance cues. Such gradient matrices are very useful tools to study 3D-cell behaviour such as invasion, vascular tube formation, neurite extension and more. Assessment of cell alignment, migration and guidance along the fibers of gradient hydrogel matrices can be performed and it would be interesting to use these matrices for modelling studies as they provide ‘quasi controlled *in vivo* 3D-systems’. Most biological tissues require a mixture of matrix-bound and soluble guidance cues that differentially contribute to cellular responses. Combinations between 3D-haptotactic and 3D-soluble gradients in hydrogels would be ideal test systems to study more complex biological behaviour such as directed cell movement and guidance. Unfortunately only one study describes such a system [8] where a gradient of a matrix-bound ECM-molecule was combined with a soluble gradient of a growth factor. Interestingly the study demonstrates that neither the ECM-gradient nor the growth factor gradient alone or in combination with a homogeneous concentration of either the growth factor or the ECM-molecule is able to induce the proper biological response. It seems that only the gradient of ECM in concert with the gradient of the growth factor seems to be able to stimulate proper cellular responses. These experiments are the first that show such clearly distinguishable effects *in vivo*. Such findings are very interesting in the light of understanding cellular guidance and tissue directionality as fundamental studies can be performed under ‘native-like’ conditions that can potentially be transformed to medically relevant situations.

**Table 1:** Gradients in 3D-hydrogel matrices.

	Gradient type/gradient production	Applications of gradients	Source
**Soluble gradients within 3D-hydrogels**	- 3D-Gradients of soluble molecules (e.g. growth factors, cytokines or hormones) within a hydrogel matrix. Gradient establishment is mostly based on diffusion. - microfluidics or macromolecular fluid devices - diffusion from chambers separated by semi-permeable membranes - microsphere-based delivery	- Gradient systems used to investigate neurite extension, angiogenesis, homing of lymphocytes- Cells cultured on top or within the hydrogel matrices - Assessment of cell alignment, migration, differentiation of stem cells - Guidance along the fibrous structures of the hydrogel matrices	[51], [77], [78], [124,125,126,127,128]
**2D-Surface gradients on top of 3D-hydrogels**	Matrix-bound (= haptotactic) gradients of biologically active molecules	- Direction of cell growth and migration to improve integration of biomedical implants and therefore facilitate healing responses - Cells cultured on top - Assessment of cell alignment, migration and differentiation	[129], [130]
**Haptotactic 3D-gradients within hydrogels**	Matrix-immobilized 3D-gradients of adhesion or extracellular matrix molecules or growth factors within a hydrogel matrix. Gradient generation: - diffusion-based or gradient mixing devices both followed by photo- or chemical immobilization	- Gradients are used to investigate 3D-cell behaviour: invasion, vascular tube formation, neurite extension Cells cultured on top or within the hydrogel matrices - Assessment of cell alignment, migration and guidance along the fibers of gradient hydrogel matrices	[8], [62], [93], [131,132,133,134]
**3D-haptotactic combined with 3D-soluble gradients in hydrogels**	Matrix-immobilized 3D-gradients of adhesion or extracellular matrix molecules or growth factors within a hydrogel matrix. Gradient generation: - diffusion-based or gradient mixing devices both followed by photo- or chemical immobilization	- Gradients are used to investigate 3D-cell behaviour: invasion, vascular tube formation, neurite extension Cells cultured on top or within the hydrogel matrices - Assessment of cell alignment, migration and guidance along the fibers of gradient hydrogel matrices	[8], [62], [93], [131,132,133,134]

## 2. Discussion and Outlook: Applications in Biomedicine

Several studies are discussed that produce soluble and matrix-bound gradients of growth factors or adhesion studies on and within 3D-hydrogel matrices. The limitations so far are that a methodology to produce the gradients and to maintain them over the entire culture period, as well as analysis of cellular behavior is still a challenge. *In vitro* 3D-gradient systems containing soluble or matrix-bound gradients cues would be very interesting to study mass transport in 3D: diffusion, convection distribution with and without barriers (e.g. different cells surrounded by basal lamina), interactions of guidance cues with the matrix and availability of matrix-bound guidance cues for embedded cells therefore directing cells towards specific targets.

**Figure 3:**
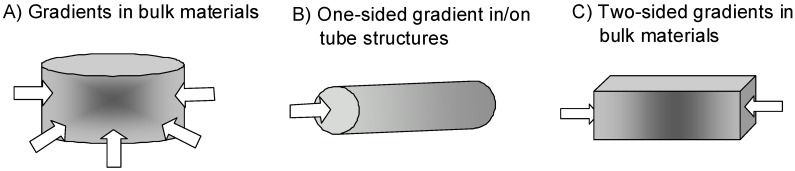
Applications for gradients in biomedicine.

Moreover, so far almost no *in vivo* study was performed, although it would be highly desirable to see if gradient guidance cues would be able to direct cellular ingrowth on/into complex biomedical implants. Some potential applications are discussed here (Figure 3): (i) Gradients in bulk materials: wound healing, (ii) One sided gradients direct cell growth towards a target: e.g. neuronal guidance, endothelial cell growth in vascular grafts; (iii) Two-sided gradients in bulk materials: potential application in critical size bone defects of cortical bone.

(A) Gradients in bulk materials: wound healing. (B) One-sided gradients in/on tubular structures direct cell growth towards a target: e.g. neuronal guidance, endothelial cell growth in vascular grafts. (C) Two-sided gradients in bulk materials: critical-size bone defects. White arrows indicate directed cellular ingrowth.

(i) Wound healing is one of the major clinical issues with increasing age of the population. Often underlying diseases such as diabetes or cardiovascular dysfunctions combined with medications induce chronic wounds that need sophisticated care and treatment [140]. Here bulk materials usable as wound filler or in combination with wound dressings providing gradients of soluble and/or matrix-bound guidance cues would be highly desirable (Figure 3A). Soluble gradients of cytokines would attract blood-derived macrophages for wound cleaning and stem cells that improve the healing response. Matrix-bound guidance cues on slow degrading fibrous matrices could overcome the inherent shortage of appropriate matrix molecules within a chronic wound as the endogenous matrix is degraded by an excess of wound-secreted proteolytic enzymes.
(ii) Another application would be to direct endothelial cells within vascular grafts or neural cells in nerve guide tubes by one-sided matrix-bound gradients of growth factors and favorable adhesion sites (Figure 3B). The motivation is that many studies have shown that endothelialization of implanted vascular grafts in humans occurs very slow and often incomplete such that the graft materials remain blood-exposed [141,142]. These materials are often thrombogenic and induce re-stenosis in about 30 % percentage of patients [143]. Gradient-type guidance of endothelial cells through the length and/or through the wall pores of the vascular graft might increase and shorten the time required for vascularization. Often traumatic experiences are accompanied with injuries in the peripheral nervous system that need to be treated in order to increase the probability of regain of function. In small injuries (< 4 mm) the injured nerve can be reconnected by direct end-to-end suturing however when larger nerve pieces are missing the remaining ends are sutured within nerve guide tubes or nerve conduits [144,145]. These polymer tubing provide guidance and protection for newly sprouting proximal nerve ends and allow reconnection with the proper target organ. In order to increase the speed and the quality of nerve regeneration it would be highly desirable to extend the studies performed by [8] that demonstrated the need of synergistic guidance cues provided by laminin-1 in combination with growth factor(s) NGF in a one-sided gradient fashion along the guidance structures.
(iii) In certain cases also two-sided gradients of guidance cues in a bulk material might be an interesting option. Cortical bone defects heal when the injury, trauma or bone loss lies within the limits of bone regeneration. Bone defects above a critical-size do not heal with bone formation instead bone is replaced by scar tissue which can not provide the load-bearing functions of bone [146]. In order to stimulate bone formation even in critical-size bone defects bone substitute materials such as hydroxyapatite, tricalcium phosphate foams, bioglasses, composite metals [146,147] and many others are filled with bone morphogenetic protein (BMP)-2. As BMP-2 is a very potent inducer of bone formation dosage and correct placing are critical issues, therefore it might be an interesting thought to introduce BMP-2 or plasmids that lead to production of BMP-2 after transfection of wound cells in a two-sided gradient manner in order to attract bone forming cells towards the center of the bone substitute material.

Summarizing, it can be stated that gradient distribution of guidance cues within or on the surface of a biomedical implant would be an interesting approach to improve biological integration into the target tissue, prolong functionality and increase the medical efficacy by attracting and directing the desired cells towards their target places on/in the medical implant.

## 3. Conclusions

Gradients within the extracellular matrix direct cell growth, migration and differentiation in the nervous- or vascular systems during development as well as in the adult where different cell types follow soluble and matrix-bound gradients of guidance cues for repair or wound healing processes. This review discusses the necessity of introducing gradients of growth factors and/or adhesion sequences for medical implants in order to improve their functionality by guiding regenerating or stem cells along the structures of the implant or improve cell penetration into 3D-porous scaffolds. As 3D-hydrogel matrices have been explored as replacements for the natural extracellular matrices they might provide the most native way to modify biomedical implants with gradients of specific adhesion molecules, growth factors or combination of both. Here three major types of gradient guidance cues within 3D-hydrogel matrices are described and discussed, namely: soluble gradients within 3D-hydrogels, immobilized 2D-gradients on top of hydrogels and immobilized 3D-gradients within hydrogels. *In vitro* studies already show improvements of tissue growth when cells orient along a gradient of one guidance cue, whereas *in vivo* several gradients need to act together in order to obtain the desired biological response. Therefore production of gradients for cell guidance within 3D-hydrogel matrices might be an interesting option not only as improvements for biomedical implants but will serve as *in vitro* culture systems to understand biological processes induced by single or superimposing gradients of biological signals.
